# Antibacterial PEEK-Ag Surfaces: Development and In Vitro Evaluation Against *Staphylococcus aureus* and *Pseudomonas aeruginosa*

**DOI:** 10.3390/jfb16100388

**Published:** 2025-10-16

**Authors:** Flávio Rodrigues, Mariana Fernandes, Filipe Samuel Silva, Óscar Carvalho, Sara Madeira

**Affiliations:** 1 Center for MicroElectroMechanical Systems (CMEMS), Mechanical Engineering, University of Minho, 4800-058 Guimarães, Portugal; 2 LABBELS—Associate Laboratory, 4800-058 Guimarães, Portugal

**Keywords:** PEEK-Ag, antibacterial, *S. aureus* and *P. aeruginosa*, dental applications

## Abstract

In the pursuit of addressing the persistent challenge of bacterial adhesion and biofilm formation in dental care, this study investigates the efficacy of electric current as an alternative strategy, specifically focusing on its application in dental contexts. Polyether ether ketone (PEEK), known for its excellent biocompatibility and resistance to bacterial plaque, was enhanced with conductive properties by incorporating silver (Ag), a well-known antibacterial material. Through systematic in vitro experiments, the effectiveness of alternating current (AC) and direct current (DC) in reducing bacterial proliferation was evaluated. The tests were conducted using two bacterial strains: the Gram-positive *Staphylococcus aureus* and the Gram-negative *Pseudomonas aeruginosa*. Various configurations, current parameters, and two different electrode configurations were assessed to determine their impact on bacterial reduction. A notable finding from this study is that alternating current (AC) demonstrates superior efficacy compared to direct current (DC). The more significant decrease in CFUs/mL for *P. aeruginosa* with AC was recorded at the current levels of 5 mA and 500 nA. In opposition, *S. aureus* exhibited the greatest reduction at 5 mA and 1 mA. This study highlights the potential of using electric current within specific intensity ranges as an alternative strategy to effectively mitigate bacterial challenges in dental care.

## 1. Introduction

One of the major clinical challenges in dental applications, all around the globe, is related to bacterial adhesion and biofilm formation [1,2]. Certainly, an imbalance in oral microbiota can impact not only overall health but also quality of life and well-being, especially in susceptible patients [3,4]. Plaque gradually develops over time as bacteria adhere to and colonize both natural and synthetic surfaces within the oral cavity, forming biofilms through biomolecular interactions [5,6]. This bacteria adhesion, particularly in hard-to-reach areas, coupled with the subsequent formation of resilient biofilms, presents obstacles to effective oral hygiene maintenance and dental restoration longevity and can lead to several infections such as dental caries (tooth decay), periodontal disease, and tooth loss, among others [7,8].

Several approaches have been explored to combat bacterial challenges in dental applications, including surface modifications, antimicrobial coatings, and novel material formulations [9]. While these strategies have shown some efficacy, their long-term effectiveness and compatibility with dental materials remain areas of concern [10,11]. Specifically, current antimicrobial strategies often fail to prevent biofilm formation in complex geometries, under dynamic oral conditions, or against resistant bacterial strains [12,13]. Within these challenges, new materials and strategies are needed [14].

PEEK exhibits excellent properties, such as high biocompatibility, low affinity to bacterial plaque, and low density (1300 kg/m^3^), which provide greater patient comfort [15]. Additionally, it is a chemically inert, hypoallergenic, and non-toxic material, capable of sterilization by radiation and heat, maintaining its mechanical properties unchanged and without causing structural damage [9,16,17]. PEEK is extensively used in various dental applications, including implants, dentures, and maxillofacial prostheses, due to its high biocompatibility and Young’s modulus comparable to that of bone. Its utilization extends to temporary abutments, fixed partial dentures, implant-supported provisional crowns, and healing caps [15,17,18,19]. Continuous modifications enhance its clinical applicability, solidifying PEEK as a non-traditional yet scientifically endorsed material within the dental domain [17]. However, even with these advantages, PEEK’s inherent resistance to bacterial colonization is insufficient under in vivo conditions, and biofilm formation remains a clinical challenge. This limitation motivates the exploration of adjunctive strategies to enhance the antibacterial properties of PEEK surfaces.

The use of electric current as an antibacterial strategy has gained attention as a promising possibility in dental care [20]. Electric current has demonstrated the potential to disrupt bacterial adhesion and biofilm formation, offering a novel approach to enhance oral hygiene and prevent dental complications [21,22]. Recent research confirms the efficacy of low voltage and electric current, even at ultra-low levels of microamperes (µA) and/or milliamperes (mA), in eradicating bacteria and biofilms [21,23,24,25,26]. Multiple studies have reported divergent outcomes regarding the antibacterial performance of alternating (AC) and direct current (DC). Thamaraiselvan et al. [27], noted that AC achieved over 98% inhibition of bacterial attachment, indicating strong biofilm suppression potential. Conversely, van der Borden et al. [28] found DC to be more efficient, producing nearly 78% detachment of *Staphylococcus epidermidis* compared to 31% with block current. Kim et al. [29] observed no significant difference among AC, DC, or combined modes when the same energy input was applied, implying that the delivered energy may be the decisive factor. More recently, Zhang et al. [30] reported over 98% bacterial inactivation with AC exposure, whereas Ruiz-Ruigómez et al. [31] confirmed that DC also exhibits strong antibacterial action across several bacterial species. Despite these advances, there is still no consensus on which current type is more efficient. The mechanisms underlying AC and DC induced bacterial reduction remain incompletely understood, emphasizing the necessity and importance of comparative studies examining their effects.

Mechanistically, electrical current application may alter bacterial membrane potential, generate reactive oxygen species (ROS), induce electrochemical gradients, and modify local pH [32,33]. Nonetheless, these mechanisms are not yet fully validated experimentally, and the specific parameters that maximize efficacy without causing tissue damage remains unclear.

In this work, PEEK was combined with silver in a precisely controlled, localized configuration, a key innovation that enables the targeted application of electrical currents. Silver not only possesses well-established antibacterial properties but, when coupled with electrical current, can exert enhanced or distinct antibacterial effects [34]. This study systematically investigates the effects of AC and DCs across a wide range of intensities, from picoamperes to milliamperes, using two electrode configurations, and evaluates their impact on *S. aureus* and *P. aeruginosa*. By integrating conductive silver into PEEK and applying localized currents, this work provides new insights into the mechanisms of bacterial inhibition and highlights the potential clinical relevance of electrically active PEEK surfaces, presenting an innovative strategy for antibacterial biomaterials.

## 2. Materials and Methods

### 2.1. PEEK Specimens

**Figure 1:**
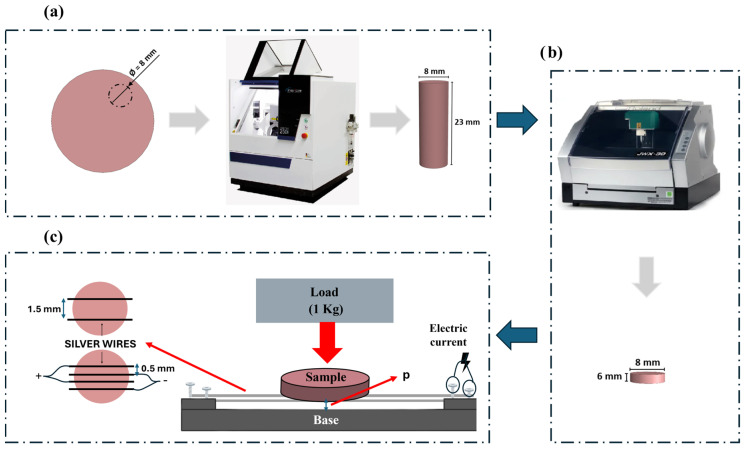
Schematic representation of the sample obtaining process: (**a**) Extraction of a cylinder from the initial block of DD PEEK Med-Gingiva, using the CNC milling machine iMes iCore 250i. (**b**) Cutting the cylinder into several thinner cylinders. (**c**) Silver wire impregnation.

PEEK samples were fabricated via CNC milling machining from DD PEEK Med-Gingiva (Dental Direct^®^, Spenge, Germany) polymer, employing two distinct CNC machines, as depicted in Figure 1a (CNC IMes icore 250i) and 1b (CNC Roland jwx-30). The primary objective was to produce cylindrical samples measuring 8 mm in diameter and 23 mm in height. Subsequently, these cylinders were further cut to achieve the final desired dimensions.

### 2.2. PEEK-Ag Specimens Production

Following the sample fabrication, the subsequent step involved establishing an electrical circuit with two distinct configurations (2-wire and 4-wire configurations) which varied in electrode distance and number, to assess antibacterial response. The samples were positioned over silver wires with 99.99 purity (Maquinouro, Porto, Portugal), which had been previously aligned and tensioned between screws, in configurations using either two or four wires. A load of 1 kg (≈10 N) was manually applied on the sample to ensure uniform contact with the wires, as schematically represented in Figure 1c. An electric current was passed through the silver wires to heat them (≈230 °C) and promote their impregnation into the PEEK. The setup was designed so that the wires (spaced at 0.5 or 1.5 mm varying with configuration) were placed at a fixed distance (p) from the base to control the impregnation depth (0.5 mm).

### 2.3. In Vitro Staphylococcus aureus and Pseudomonas aeruginosa Biofilms

**Figure 2:**
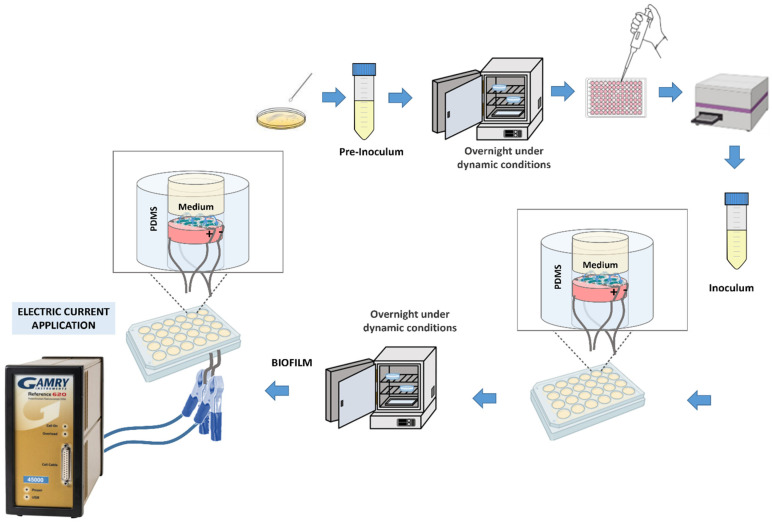
Schematic representation of biofilm development and electric current application.

For the preparation of the bacterial pre-inoculum, a single colony of *S. aureus* (CIP 76.25) *and P. aeruginosa* (CIP 76.110) was isolated from an agar plate, which was then cultured in a Falcon containing 30 mL of nutrient medium, TSB (Tryptone Soya Broth), at 37 °C overnight in an incubator (Raypa^®^, Incuterm, Barcelona, Spain) with agitation at 120 rpm (IKA-VIBRAX-VXR) until reaching the stationary growth phase of bacterial growth. To estimate the population of bacterial cells, an optical density (OD) reader (BioTek Epoch™ Microplate Spectrophotometer, Agilent Technologies, Santa Clara, CA, USA) at 600 nm was used, with Gen5 software, measuring three wells with 100 µL each. The obtained values were adjusted to an optical density of 0.125, corresponding approximately to 1.25 × 10^7^ CFU/mL. From the obtained inoculum, 1 mL was withdrawn and placed in contact with each sample in 24-well plates. Subsequently, the plates containing the samples in contact with the bacteria were placed in the incubator at 37 °C for 24 h with agitation. Figure 2 illustrates a schematic representation of the process. Both *S. aureus* and *P. aeruginosa* were tested independently.

### 2.4. PEEK-Ag Electric Current Application

**Figure 3:**
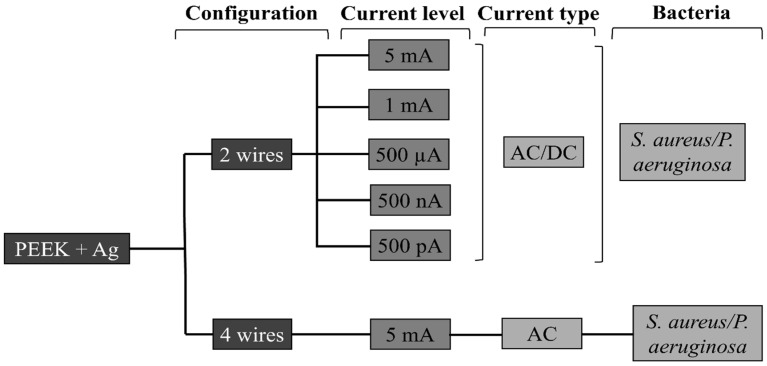
Schematic representation of variables in study.

The electrical current was applied to the samples after 24 h in contact with bacteria. For the samples subjected to stimulation, the intensity and type of current (Direct Current DC or Alternate Current AC) applied were varied. For electric current application a potentiostat (Gamry® 600+, Gamry Instruments, Warminster, PA, USA) was used. The current intensities tested in this study were: 5 mA, 1 mA, 500 µA, 500 nA, and 500 pA. The current was applied for 10 min to each of the samples [24]. Figure 3 displays a representation of the study variables. It should be noted that all AC and DC levels were initially tested using the 2-wire configuration. After identifying the best condition regarding CFUs reduction (AC 5 mA), this single condition was then tested on both 2- and 4-wire configurations to compare different electrode configurations.

### 2.5. Quantitative Evaluation of Adhered Bacteria

The effect of electric current application under different parameters was evaluated using a colony-forming unit (CFU/mL) assay to assess bacterial presence, adhesion, and viability on the sample surfaces. After the 10 min electrical stimulation period, the fluid/medium in the wells along with the samples was discarded, and each sample was placed in a Falcon containing 6 mL of PBS (Phosphate-Buffered Saline). Subsequently, these tubes were subjected to ultrasonic bath treatment (Branson 3510-DTH Ultrasonic Cleaner, Branson, Danbury, CT, USA) for 10 min, followed by vertexing (Heidolph™ Reax Top Vortex Mixer, Schwabach, Germany) for 1 min each, to dislodge the bacteria adhered to the surface. In the next step, serial dilutions were performed by adding 90 µL of PBS to 5 wells for each sample. In the first well of each sample, 10 µL of the solution from the Falcons were added, and dilution was carried out from there. Once dilutions were completed, 5 µL of all dilutions were plated using the drop method, with two replicates per sample, on TSA agar plates (Trypticase Soy Agar), and then incubated at 37 °C for 24 h under static conditions in the incubator.

### 2.6. Surface Characterization

The average surface roughness of the produced samples was measured using a surface profilometer (Mitutoyo Surftest SJ-210 Series, Mitutoyo, Kawasaki, Japan). This highly sensitive instrument features a diamond-tipped spherical probe with a diameter of 2 µm and applies a force of 0.75 N/m to the sample.

Measurements were conducted on 3 samples of each configuration (2 and 4 wires), with three readings per sample taken perpendicular to the impregnated wires, using a probe displacement of 0.30 mm and a speed of 0.5 mm/s. Additionally, the average surface roughness of PEEK samples without silver wires was measured for comparative purposes. The surface roughness was determined through arithmetic mean.

To assess antibacterial effect after electric current application, PEEK-Ag samples underwent fixation in a 2.5% glutaraldehyde solution for 4 h at 4 °C. Following fixation, they were sequentially washed in PBS and sterile water (10 min per immersion). Subsequently, a dehydration process was initiated using graded ethanol solutions (30%, 50%, 70%, 80%, 90%, and 100%; 30 min per series). The dehydrated specimens were then subjected to the critical point drying method, sputter-coated with gold, and observed using scanning electron microscope (JEOL JSM-6010LV, Akishima, Japan).

### 2.7. Temperature Evaluation

Considering both human body applications and that the application of electric current could lead to temperature variations, this variable was measured during the tests. An IR thermographic camera (FLIR^®^ Ax5 series, FLIR Systems, Wilsonville, OR, USA) was used to acquire the temperature in disks’ surfaces from 0 to 10 min every 150 s. The data were then analyzed with FLIR^®^ Research IR (Wilsonville, OR, USA) software.

### 2.8. Statistical Analysis

For statistical analysis, GraphPad^®^ Prism version 10.0.0 for Windows (GraphPad^®^ Software, Boston, MA, USA) was used. Initially, as the number of samples was less than 50, the Shapiro–Wilk test was performed to check the normality of the data. Since the data did not follow normal distribution, non-parametric tests were chosen, specifically the Kruskal–Wallis test, which is used when comparing three or more independent groups, as in this case. For multiple comparisons between groups, Dunn’s test was used. Additionally, in some situations where only two conditions were being compared, the Mann–Whitney test was used. The tests were conducted with a confidence level of 95%, indicating that the selected significance level (α) corresponds to 0.05. For tests of normality, the null hypothesis is H0: The data follows normality. If the *p*-value is less than or equal to the imposed significance level (*p*-value ≤ α), there is statistical evidence to reject the null hypothesis, meaning the data do not follow a normal distribution. Conversely, if the *p*-value is greater than the significance level (*p*-value > α), the data follows a normal distribution. In multiple comparisons, the null hypothesis is H0: There are no differences between conditions. If the *p*-value ≤ α, the null hypothesis is rejected, indicating statistically significant differences between conditions.

## 3. Results

This study explored the potential of applying electric current to address *Staphylococcus aureus* and *Pseudomonas aeruginosa* biofilms on PEEK surfaces. The experimental design incorporated a silver pathway for current delivery (electrode) to evaluate bacterial adhesion to the samples surfaces after various tests with different types of current and intensities. The findings provide new insights into biofilm treatment strategies, particularly in identifying optimal electric currents for targeting biofilms on PEEK surfaces

### 3.1. PEEK-Ag Specimens’ Characterization

Table 1 presents the average roughness (Ra) values obtained for PEEK and the two PEEK-Ag configurations tested in vitro in this work (PEEK, PEEK-Ag-2 wires, and PEEK-Ag-4 wires). According to the literature Quirynen et al. (1996) [35], average roughness (Ra) values above approximately 0.2 μm promote bacterial adhesion. Considering the highest roughness value obtained (0.027 ± 0.012 µm), it can be concluded that this is lower than the known surface average roughness value (Ra ≈ 0.2 μm) that affects microbiological surface load. Therefore, it can be considered that roughness is not a factor significantly contributing to bacterial adhesion to the surface.

### 3.2. Temperature Evaluation During Electric Current Application

The oral environment, including soft tissues and bone, is highly sensitive to temperature changes and maintaining appropriate temperature levels is crucial during dental procedures to prevent damage to oral soft tissues [36]. Oral tissues are naturally maintained at body temperature (≈37 °C) and substantial deviations could impact the soft tissues, osseointegration and also the patient comfort.

Despite there is a non-defined range of safety temperatures, Zach and Cohen’s [37] in vivo study on monkeys showed that an increase of 5.5 °C caused already damage in pulp tissue. In what concern to alveolar bone, an increase of 10 °C caused bone resorption as studied by Eriksson and Albrektson [38].

Table 2 depicted the results of the present study, demonstrated that the temperature increase was minimal across all tested conditions, highlighting the thermal stability of the materials and their suitability for oral implantation. The maximum temperature recorded was 1.7 °C when a 20 mA AC was applied. This current was tested for temperature evaluation and was not used in the bacterial experiments, as it is four times higher than the maximum current level applied in regular tests (5 mA). The 20 mA condition was included to assess whether higher current intensities could cause a significant temperature rise that might be harmful to biological tissues. The results demonstrated that even at this elevated current, the temperature increase remained minimal, confirming that the applied current levels are safe for biological applications.

Interestingly, the data revealed no clear correlation between the type of current (AC or DC) and the observed temperature changes, nor between the current levels and temperature elevation. These results suggest that the tested materials maintain stable the system PEEK-Ag temperature under both ACs and DCs, even at elevated levels. Overall, the temperature increases observed remained well within the safety thresholds established in the literature for biological tissues [39]. This is a significant finding, as it underscores the thermal compatibility and safety of the tested materials for use in oral environments.

### 3.3. Effect of Electric Current Application on Bacterial Adhesion to PEEK-Ag Surface

The present study aimed to assess the influence of different types (Ac and DC) and levels of electric current on bacterial adhesion to PEEK-Ag surfaces. *Staphylococcus aureus* (Gram-positive) and *Pseudomonas aeruginosa* (Gram-negative) were the bacteria used, and the results demonstrated that bacterial attachment was significantly affected by the application of electric current. Differences were observed between alternating current (AC) and direct current (DC) and in different levels of each type of current. In general, AC was notably more effective in reducing bacterial adhesion, particularly at higher intensities, whereas DC exhibited a more limited impact under comparable conditions. However, no consistent trend in bacterial adhesion was observed across different intensity levels within the same type of current.

**Figure 4:**
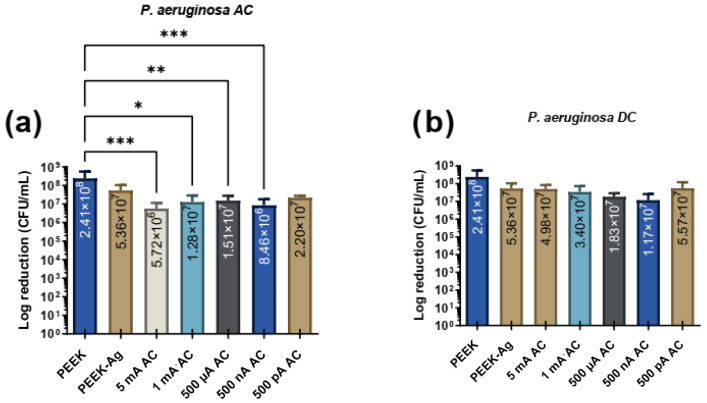
Bacterial adhesion (CFU/mL) of *P. aeruginosa* on the tested surfaces after 24 h in contact with specimen’s surface and then treated with electrical current application with (**a**) AC and (**b**) DC. In both figures, * corresponds to *p* < 0.1, ** is *p* < 0.01, *** is *p* < 0.001.

Figure 4 presents the effects of different intensities of AC and DC with different levels of intensities for *P. aeruginosa* adhesion. The study includes the PEEK specimens control group, consisting of samples without current application, as well as the PEEK-Ag specimens group, which represents samples with silver but no applied current. Additionally, it includes PEEK-Ag samples exposed to varying current intensities, ranging from 5 mA to 500 pA.

Figure 4 shows that under AC elctric current application, a notable reduction in bacterial CFUs/mL was observed. Comparing the control PEEK group to the PEEK-Ag group, a reduction of approximately 0.7 log (close to 1 log) was achieved. With the application of 5 mA, type AC, bacterial CFUs/mL were reduced by nearly 2 logs, indicating a significant antimicrobial effect. At lower current levels, including 1 mA, 500 µA, and 500 nA, reductions of approximately 1 log were observed. Notably, the 500 nA condition resulted in a reduction exceeding 1 log but not reaching 2 logs, suggesting enhanced effectiveness compared to other lower current levels. Statistical analysis showed significant differences between the control group and all tested AC levels, except for the lowest current level (500 pA), which did not produce a statistically significant reduction.

In contrast, DC application resulted in CFU/mL reductions of approximately 1 log across nearly all tested current levels, with the 500 nA condition yielding the lowest bacterial counts. However, statistical analysis revealed no significant differences between the control (PEEK) and any of the DC levels, indicating that the observed reductions were not robust enough to achieve statistical significance. When comparing the effects of AC and DC stimulation, AC proved to be more effective in reducing bacterial CFUs/mL. AC conditions demonstrated statistically significant reductions at multiple current levels, whereas DC did not achieve significance at any level. Furthermore, neither AC nor DC showed a linear correlation between increasing current levels and bacterial reduction; however, AC consistently exhibited a stronger antimicrobial effect across the tested conditions.

**Figure 5:**
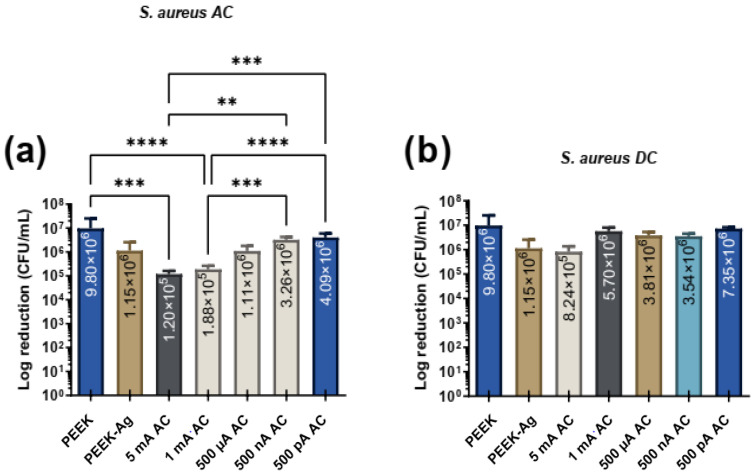
Bacterial adhesion (CFU/mL) of *S. aureus* on the tested surfaces after 24 h in contact with specimen’s surface and then treated with electrical current application with (**a**) AC and (**b**) DC. In both figures, ** corresponds to *p* < 0.01, *** is *p* < 0.001 and **** is *p* < 0.0001.

Figure 5 presents the effects of different intensities of AC and DC with different levels of intensities for *S. aureus* adhesion.

Under AC application, significant bacterial reductions were observed at higher current levels, with both 5 mA and 1 mA achieving reductions of nearly 2 logs. At lower current levels, reductions were present but did not reach statistical significance. For DC stimulation, no statistically significant differences were found across all tested current levels. However, the maximum reduction in CFUs/mL under DC conditions was observed at 5 mA.

Similarly to *P. aeruginosa*, no linear correlation between lower current levels and greater bacterial reduction was observed for *S. aureus*. When comparing the two bacterial species, the greatest reduction was achieved for *S. aureus* under AC stimulation, suggesting that *S. aureus* appears to be slightly more susceptible to AC treatment than *P. aeruginosa*. In contrast, under DC stimulation, the differences between the two bacteria were insignificant, indicating similar levels of resistance to DC across the tested conditions. These results further highlight the potential of AC stimulation, particularly at higher current levels (mA), as a more effective antimicrobial approach for both bacterial species.

For both bacteria species the treatment with ACs showed, more promising results when compared with DCs which is in line with some previous research [40,41,42].

Several factors could contribute to the bacterial reduction observed in this study. These factors may include effects directly associated with the application of electric current, such as damage to bacterial membranes, and indirect influences like local changes in pH and the possible formation of reactive oxygen species (ROS) [43,44]. When an electric current is applied, electrochemical reactions at the PEEK–Ag surface could produce oxidative species and shift the acidity of the surrounding medium, creating stresses on bacterial cells [45]. The electric field might also interfere with the transmembrane potential of bacteria, increasing membrane permeability and enabling leakage of intracellular content [46,47]. Moreover, current flow could enhance the release or mobility of silver ions, which are antibacterial [48]. While these mechanisms are plausible considering the observed antibacterial effects, this study does not provide direct measurements. In future work should include fluorescence-based assays to assess membrane integrity, quantitative ROS measurements, and direct characterization of ion release under applied electric current to validate these hypotheses.

**Figure 6:**
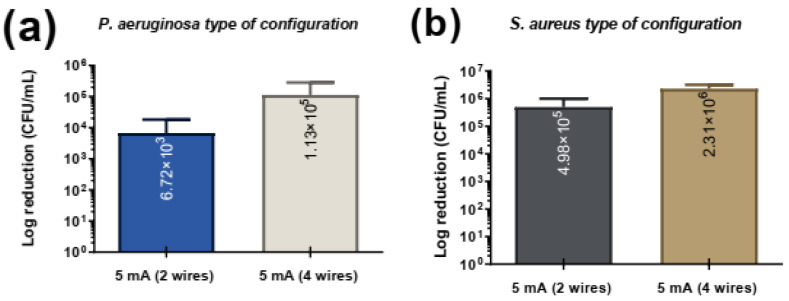
Comparison of bacteria adhesion as a function of the type of configuration in (**a**) *P. aerugionosa* and (**b**) *S. aureus*.

An additional experiment was conducted to evaluate the effect of electrode configuration on bacterial CFUs/mL under 5 mA of current for both *Pseudomonas aeruginosa* and *Staphylococcus aureus* (Figure 6). Two configurations were tested: a 2-wire setup (greater distance between electrodes 1.5 mm) and a 4-wire setup (shorter distance between electrodes 0.5 mm). For both bacterial species, the 2-wire configuration achieved greater bacterial reduction compared to the 4-wire configuration.

The observed greater bacterial reduction in the 2-wire configuration can be attributed to differences in electric field distribution caused by electrode spacing. A larger distance between electrodes likely resulted in a more uniform and widespread electric field across the sample, effectively covering a larger volume and exposing more bacteria to the current. In contrast, the 4-wire configuration, with its shorter inter-electrode distance, may have created a more localized and less effective electric field, reducing its overall efficacy in eliminating bacteria throughout the sample. This phenomenon is consistent with prior research. Studies have shown that increased electrode spacing can improve the distribution of the electric field, enhancing microbial inactivation [49,50]. For example, research on microbial fuel cells has demonstrated that greater electrode spacing enhances performance by reducing internal resistance and creating a more effective electric field distribution, which supports better biogas production [50]. Additionally, a study on pulsed electric fields indicates that electrode geometry and spacing play critical roles in determining field distribution and microbial inactivation efficiency [49]. These findings underscore the importance of optimizing electrode configurations, with greater inter-electrode distances offering potential advantages in enhancing the antimicrobial efficacy of electrical stimulation.

**Figure 7:**
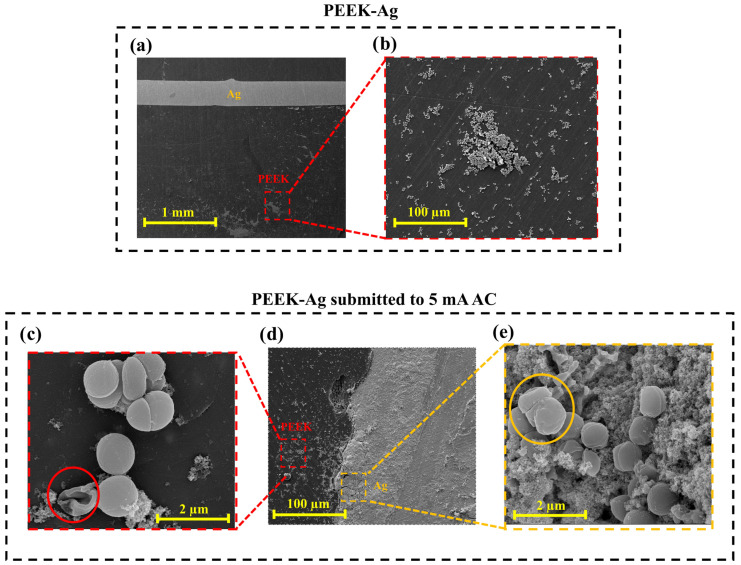
SEM micrographs of PEEK–Ag samples colonized with *S. aureus*: (**a**) control sample showing PEEK and Ag wire regions (×500), (**b**) magnified view of PEEK region (×2000), (**c**) PEEK region of sample subjected to 5 mA AC for 10 min (×2000), (**d**) PEEK–Ag sample after current application (×500), and (**e**) Ag region of the same sample (×2000).

Figure 7 shows SEM micrographs of the PEEK–Ag samples, including the control specimen, which was not exposed to electric current, and the sample subjected to 5 mA AC, identified as the most effective antibacterial condition. In Figure 7a, two well-defined regions can be observed, corresponding to the PEEK substrate and the Ag wire. It is evident that the area closer to the Ag wire presents fewer adhered bacteria compared to regions farther away, suggesting that the presence of silver exerts a local antibacterial influence. The PEEK region, in contrast, shows a higher degree of bacterial colonization, which is consistent with the CFU results indicating greater bacterial growth on the control PEEK surface. The magnified image in Figure 7b further confirms this observation, showing clusters of *S. aureus* cells on the PEEK surface, with fewer colonies detected in zones adjacent to the Ag wire, possibly due to the diffusion of silver ions or localized antimicrobial effects.

The sample exposed to 5 mA AC is shown in Figure 7d, where the two regions of PEEK and Ag wire are again visible. The corresponding magnified images (Figure 7c,e) represent the PEEK area (red box) and the Ag area (yellow box), respectively. In both magnifications, bacterial cells exhibit clear signs of membrane rupture and deformation, indicating that the applied alternating current can directly compromise bacterial membrane integrity, ultimately leading to cell death [44,51,52]. In the Ag region (Figure 7e), a thin layer of oxide can also be observed along with bacterial debris. These oxides are likely formed as a secondary effect of current application and may enhance antibacterial performance by promoting silver ion release and adding intrinsic antimicrobial properties [53,54]. Overall, the comparison between the control and the electrically stimulated samples suggests a synergistic antibacterial mechanism involving both the direct action of the electric current, which disrupts bacterial membranes, and the indirect effects associated with oxide formation and silver ion release. Together, these factors contribute to the pronounced reduction in bacterial adhesion and the visible morphological damage observed on the 5 mA AC-treated PEEK–Ag surfaces.

## 4. Conclusions

In conclusion, the results suggest that, overall, alternating current demonstrated more favorable outcomes than direct current. For AC, the most significant reduction in CFUs/mL for *P. aeruginosa* was observed at current levels of 5 mA and 500 nA. In contrast, for *S. aureus*, the best reduction occurred at 5 mA and 1 mA. For DC, no significant differences were observed between the different current intensities. Regarding the electrical configuration, the 2-wire setup was found to be more effective, leading to a greater reduction in bacterial counts compared to the 4-wire configuration. The bacterial species also influenced the results, with *S. aureus* showing higher susceptibility to the applied current, resulting in a more substantial reduction in CFUs/mL than *P. aeruginosa*. Overall, electric current appears to be a promising alternative antimicrobial strategy, particularly when applied as alternating current (AC). However, further studies are required to confirm these findings and optimize the parameters for practical applications.

## Figures and Tables

**Table 1:** Average Roughness (Ra) of tested samples.

Configuration	Mean ± SD (µm)
PEEK	0.016 ± 0.001
PEEK-Ag-2 wires	0.018 ± 0.004
PEEK-Ag-4 wires	0.027 ± 0.012

**Table 2:** Temperature variation according to sample configuration, current type and current levels applied.

Configuration	Current Type	Current Level	Temperature Variation
2 wires	AC	20 mA	1.7 °C
		5 mA	0.4 °C
		500 µA	0 °C
		500 nA	0.1 °C
		500 pA	0.2 °C
	DC	5 mA	0.2 °C
		500 µA	0 °C
		500 nA	0 °C
		500 pA	0 °C
4 wires	AC	5 mA	0.4 °C

## Data Availability

The original contributions presented in this study are included in the article. Further inquiries can be directed to the corresponding author.
